# Enhanced visible light photocatalysis through fast crystallization of zinc oxide nanorods

**DOI:** 10.3762/bjnano.1.3

**Published:** 2010-11-22

**Authors:** Sunandan Baruah, Mohammad Abbas Mahmood, Myo Tay Zar Myint, Tanujjal Bora, Joydeep Dutta

**Affiliations:** 1 Center of Excellence in Nanotechnology, School of Engineering and Technology, Asian Institute of Technology, Klong Luang, Pathumthani 12120, Thailand. Phone: +66 2 524 5680, Fax: +66 2 524 5617

**Keywords:** defects, nanoparticle, nanorod, photocatalysis, pollutant, ZnO

## Abstract

Hydrothermally grown ZnO nanorods have inherent crystalline defects primarily due to oxygen vacancies that enhance optical absorption in the visible spectrum, opening up possibilities for visible light photocatalysis. Comparison of photocatalytic activity of ZnO nanorods and nanoparticle films on a test contaminant methylene blue with visible light irradiation at 72 kilolux (klx) showed that ZnO nanorods are 12–24% more active than ZnO nanoparticulate films. This can be directly attributed to the increased effective surface area for adsorption of target contaminant molecules. Defects, in the form of interstitials and vacancies, were intentionally created by faster growth of the nanorods by microwave activation. Visible light photocatalytic activity was observed to improve by ≈8% attributed to the availability of more electron deficient sites on the nanorod surfaces. Engineered defect creation in nanostructured photocatalysts could be an attractive solution for visible light photocatalysis.

## Introduction

Photocatalysis is a light induced catalytic process whereby photogenerated electron-hole pairs in a semiconductor undergo redox reactions with molecules adsorbed onto the surface, thereby breaking them into smaller fragments. Photocatalysis with metal-oxide-semiconductor nanostructures has been an area of intense research over the last couple of decades with titania (TiO_2_) receiving the most attention [1–4]. Optical absorption in the ultraviolet region (peaking around 220 nm) [5] and low photoefficiency [6–7] are factors that deter the wide scale use of TiO_2_ for photocatalytic activities under sunlight. Zinc oxide (ZnO), with a high surface reactivity owing to a large number of native defect sites arising from oxygen nonstoichiometry, has emerged to be an efficient photocatalyst material compared to other metal oxides [8–10]. ZnO exhibits comparatively higher reaction and mineralization rates [11] and can generate hydroxyl ions more efficiently than titania (TiO_2_) [12]. Surface area and surface defects play an important role in the photocatalytic activity of metal-oxide nanostructures, as the contaminant molecules need to be adsorbed on to the photocatalytic surface for the redox reactions to occur. The higher the effective surface area, the higher will be the adsorption of target molecules leading to better photocatalytic activity. One dimensional nanostructures, such as nanowires and nanorods, offer higher surface to volume ratio compared to nanoparticulate coatings on a flat plate [13]. The design of transition metal-oxide mesostructures has also been an area of interest for researchers [14–15] owing to high porosity of such structures. The effective surface area (adsorbed amount of target molecules) and the diffusivity are important indexes to gauge photocatalytic activity [14]. Doping of metal oxides with metals and/or transition metals creates quasi-stable energy states within the band gap (surface defects) [16], thereby affecting the optical and electronic properties [17]. Increased electron trapping due to higher defect sites leads to enhancement in the photocatalytic efficiency. This increase in photocatalytic efficiency is possible provided the electron-hole pair recombination rate is lower than the rate of electron transfer to adsorbed molecules. There are reports on the enhancement of visible light absorption in ZnO by doping with, e.g., cobalt (Co) [18], manganese (Mn) [19], lead (Pb) and silver (Ag) [16], etc. Photocatalytic activity comparable to doped ZnO was also observed with engineered defects in ZnO crystals achieved by fast crystallization during synthesis of the nanoparticles [20].

Results from photocatalysis experiments carried out using ZnO nanoparticles prepared through a slow growth process (3 h hydrolysis at 60 °C) and rapid crystallization (7 min under microwave irradiation) have already been reported in a previous publication [20]. A higher optical absorption in the visible region was observed in this case. The faster degradation of methylene blue (MB) in the presence of nonstoichiometric crystallites of ZnO prepared through fast crystallization can be attributed to quasi-stable surface states that enhance the photocatalytic activity. Time correlated single photon count spectroscopy (TCSPCS) carried out on conventionally synthesized and rapidly crystallized nanoparticles clearly demonstrated that a higher rate of injection of photoexcited electrons occurs in the molecules adsorbed on the surface of defect engineered nanoparticles [21]. Upon excitation of ZnO nanoparticles with high-energy laser pulses at 375 nm in the presence of MB molecules, a quick decay in photoluminescence in the fast-crystallized particulate samples as compared to the conventionally hydrolyzed ones was observed. The spectroscopic results support the observation that surface defect states are created when the ZnO crystals are grown rapidly, which facilitates surface reactions such as photocatalysis. Even though the ZnO nanoparticles could efficiently carry out photocatalytic degradation of MB, the removal of the 5–7 nm sized particles after the completion of the photocatalytic reactions is cumbersome. This necessitates the use of supports for the photocatalysts. In this work we have used glass slides as the support for ZnO nanorod photocatalysts. When affixed on to a support, ZnO nanorods offer higher surface to volume ratio compared to nanoparticulate films, allowing higher adsorption of the target molecules [20].

Nonstoichiometric growth of the ZnO crystal was achieved by microwave heating that offers numerous advantages over conventional autoclave heating, such as rapid rise to crystallization temperatures, homogenous nucleation and fast supersaturation by rapid dissolution [22–27]. In this work a study is conducted on the improvement of visible light photocatalytic degradation of a model organic dye, methylene blue, with ZnO nanorods grown by a rapid growth process using microwave irradiation.

## Results and Discussion

**Figure 1:**
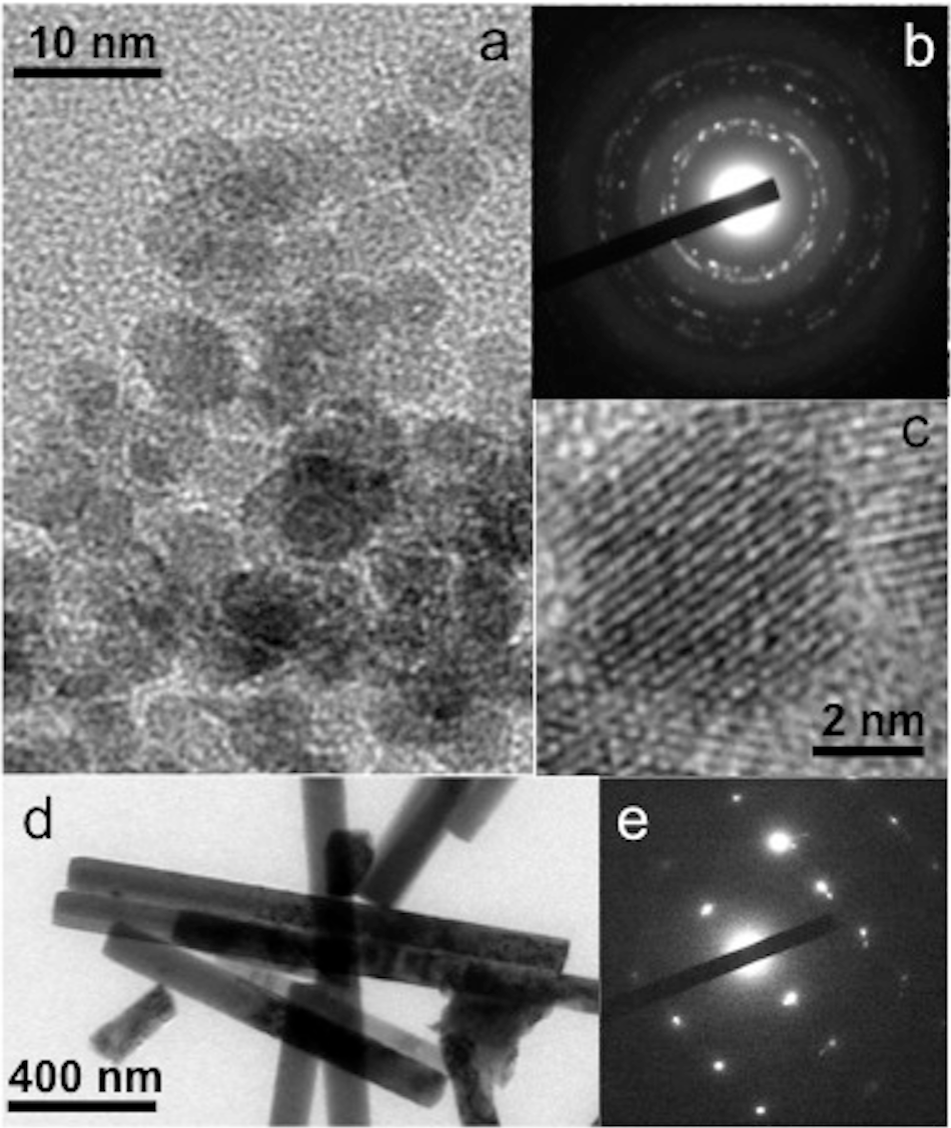
(a) Low-resolution TEM micrograph of ZnO nanoparticles, (b) electron diffraction pattern of the ZnO nanoparticles, (c) high-resolution TEM micrograph of a ZnO nanoparticle showing the lattice fringes, (d) low-resolution TEM micrograph of ZnO nanorods, and (e) electron diffraction pattern taken on a single ZnO nanorod.

The ZnO nanoparticles obtained through the sol–gel synthesis (see section Experimental) are shown in Figure 1a and Figure 1b as transmission electron microscopy (TEM) micrographs. The low-resolution TEM micrograph exhibits spherical nanoparticles with diameters of 5 to 7 nm in size (Figure 1a). Measurements of the lattice fringe widths on the high-resolution TEM micrographs (see Figure 1c) confirm the wurtzite structure of the zinc oxide crystallites. Fringe widths of 0.28 nm, 0.16 nm and 0.19 nm measured on different images show the dominance of the (100), (110) and (102) planes. The electron diffraction pattern shown in Figure 1b also confirms the crystallinity of the ZnO nanoparticles. A TEM micrograph of ZnO nanorods collected from the glass substrate is shown in Figure 1d. The diffraction pattern taken on a single rod is shown in Figure 1e demonstrating a single crystalline structure.

**Figure 2:**
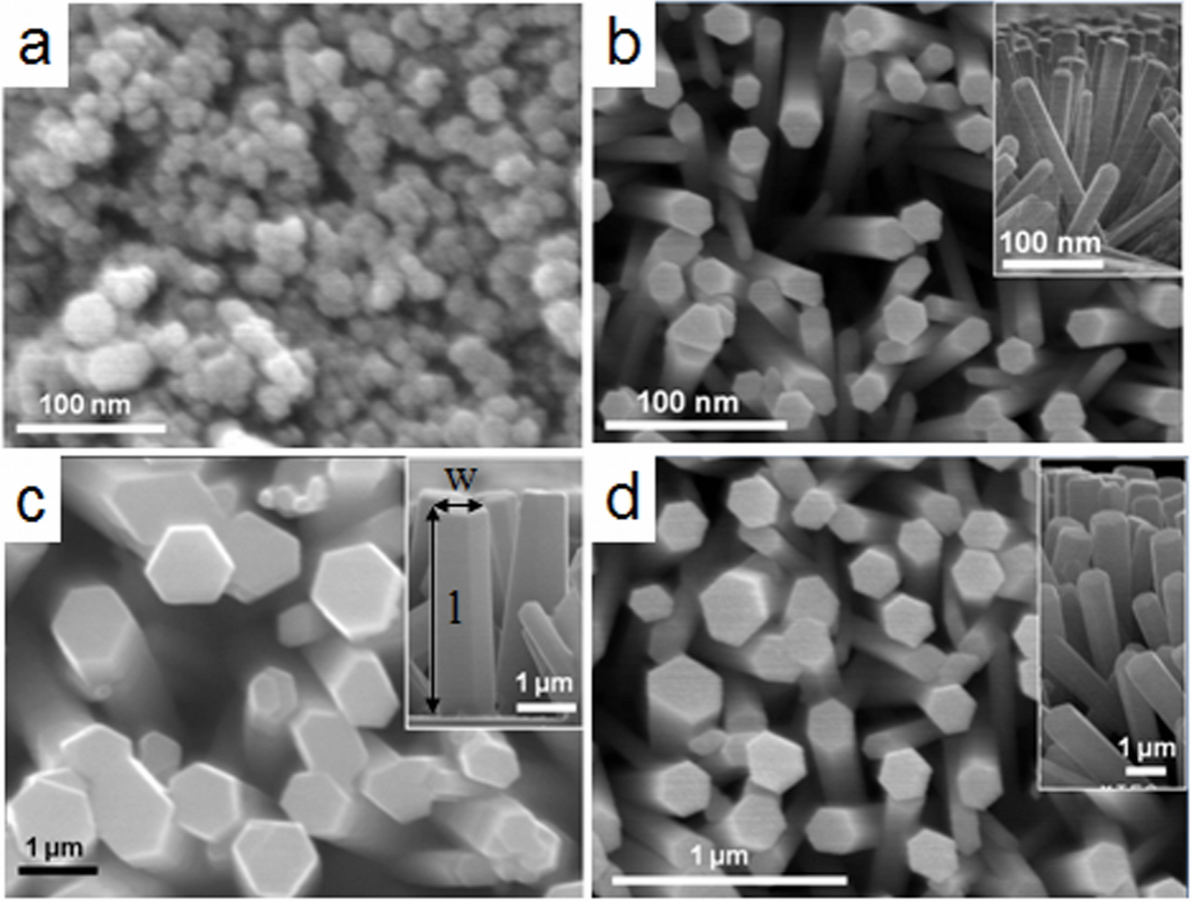
Scanning electron micrograph of (a) ZnO nanoparticle thin film on glass substrate, (b) Sample 1 (0.1 mM), inset: cross-sectional view, (c) Sample 2 (1.0 mM), inset: cross-sectional view, and (d) Sample 3 (10.0 mM), inset: cross-sectional view.

The width, length and density of the nanorods on the substrates are dependent on the synthesis conditions, such as, seeding of the substrates, concentration of precursors in the growth solution, as well as the duration of hydrothermal process [27–29]. The effective surface area available for dye adsorption is a function not only of the thickness and length of the nanorods, but also the density of the rods covering the substrate. The average exposed surface area was approximated from measurements using the scanning electron micrographs (Figure 2) considering regular hexagonal ZnO nanorods. The free surface area available for dye adsorption was estimated with the expression

[1]
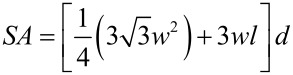


where *SA* is the total surface area, *w* is the average width, *l* the average length and *d* the number density of the ZnO nanorods on the substrate. In Table 1 the estimated values of the exposed surface area available for adsorption of the dye molecules in the different samples are presented.

**Table 1:** Estimated effective area of ZnO nanorod surfaces on substrates of size 1 × 3 cm, grown at different reactant concentrations during the hydrothermal growth process at 90 °C with equimolar concentrations of zinc nitrate and hexamine in the starting aqueous solution.

Parameter	Sample 1	Sample 2	Sample 3

Growth concentration	0.1 mM	1.0 mM	10.0 mM
Average width of nanorod *w* (nm)	20	200	1000
Average length of nanorod *l* (nm)	150	1800	5000
Area of each nanorod (µm^2^)	0.00952	1.132	16.3
Number density of the nanorods on substrate (µm^−2^)	1200	14	0.8
Total effective surface area on a substrate of size 1 × 3 cm (cm^2^)	34.27	47.54	39.12

**Figure 3:**
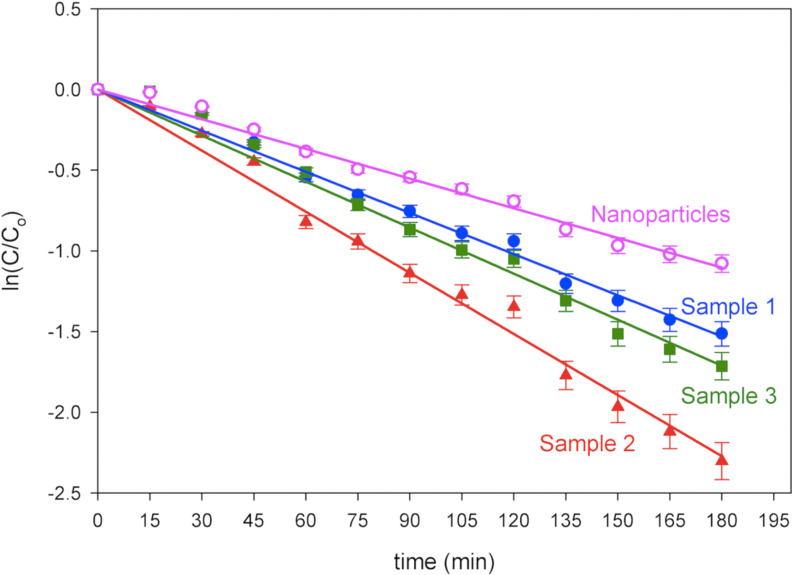
Degradation of methylene blue as a function of ln(*C*/*C*_0_) versus the time of exposure to visible light on the ZnO nanoparticle film and the nanorods with different dimensions.

Figure 3 shows the photocatalytic results comparing the ZnO nanoparticle film and the nanorods of different sizes and density (thereby offering different surface to volume ratios) on glass substrate (3 cm^2^). The photocatalytic degradation of MB could be fitted using Equation 2 and the apparent rate constants (*k* = *ab*) were calculated from the linear curves using Equation 3:

[2]
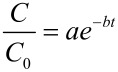


[3]
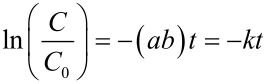


The nanoparticle film demonstrated minimum photocatalytic activity as expected owing to lower surface (*SA* ≈ 6 cm^2^) exposed to the contaminant molecules [10]. After 180 min of visible light illumination at 72 klx, the nanoparticulate thin film showed minimum activity (degradation efficiency of 64% after 180 min; *k* = 0.005993 min^−1^). Sample 2 with the maximum surface area (47.54 cm^2^) gave the highest activity (degradation efficiency of 90%; *k* = 0.012792 min^−1^) and sample 1 with the minimum surface area among the nanorod samples (34.27 cm^2^) had the lowest activity (degradation efficiency of 78%; *k* = 0.008412 min^−1^).

**Figure 4:**
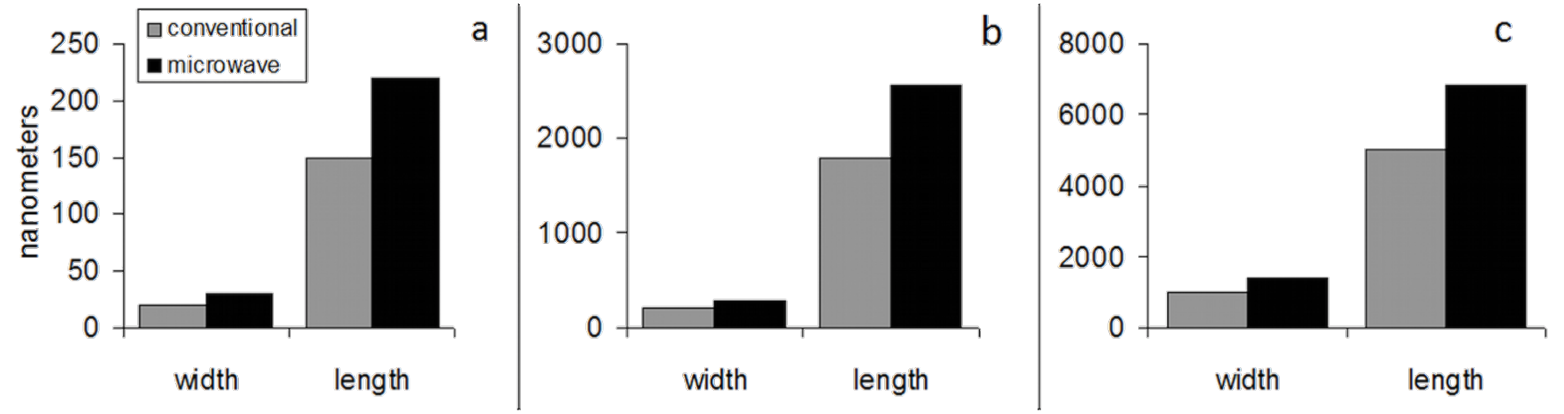
Increase in width and length resulting from fast crystallization by the use of microwave irradiation after 10 h growth at different growth concentrations: (a) 0.1 mM, (b) 1.0 mM, and (c) 10.0 mM.

As with the ZnO nanoparticles [17–18], an increase in the density of vacancies and interstitial defects in the nanorod crystals were obtained through accelerated crystallization using microwaves and subsequent fast quenching reactions. Apart from the oxygen nonstoichiometry, microwave induced growth also reduces the growth time. A comparison of the ZnO nanorod growth using the conventional process and through fast crystallization (with microwave irradiation) is shown in Figure 4. The width and length of the nanorods were measured after 5 h growth with starting solutions of 0.1 mM, 1.0 mM and 10.0 mM zinc nitrate hexahydrate and hexamethylenetetramine at a temperature of 90 °C. An increase in size was observed for the samples prepared by microwave excitation indicating a faster crystallization process. About 40 to 50% increase in width of the nanorods was noted for the samples prepared by microwave induced hydrolysis reactions. Similarly, an increased length of ZnO nanorods by 36 to 66% was obtained for different growth concentrations of the reactant solution.

**Figure 5:**
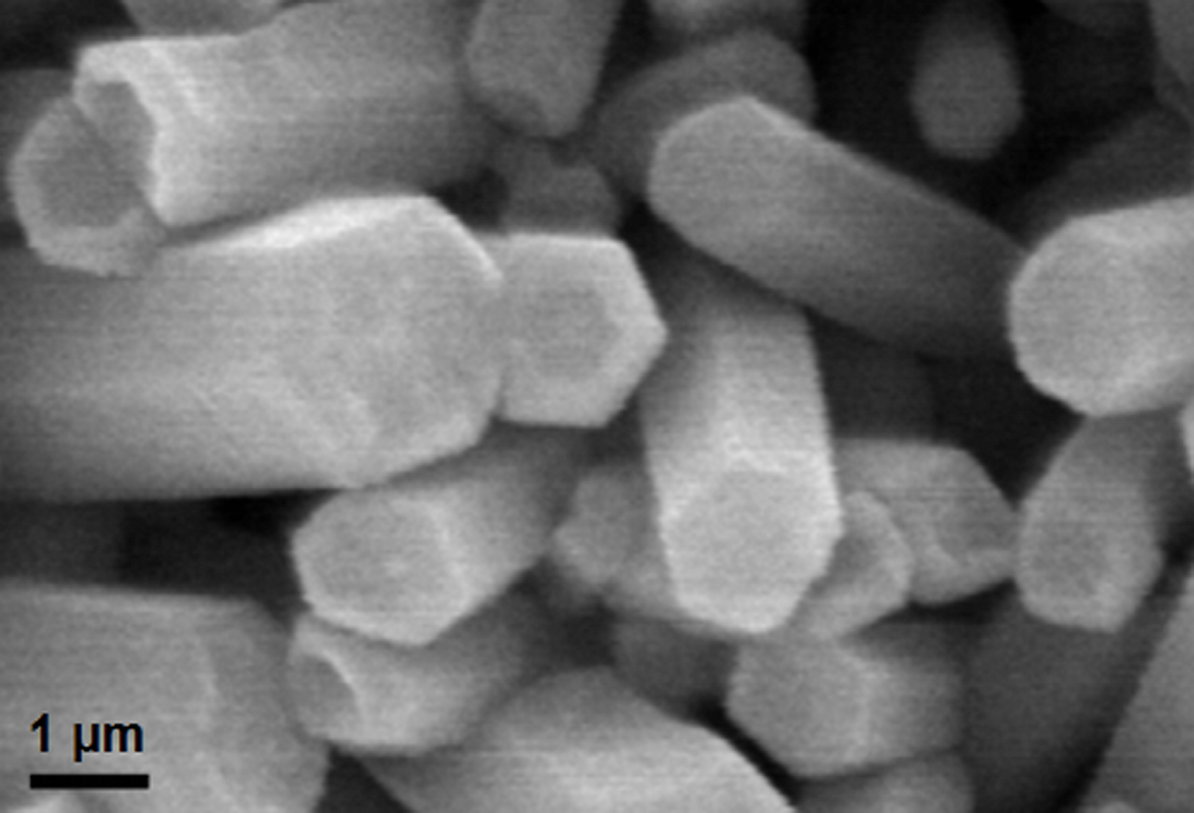
A typical scanning electron micrograph showing the ZnO nanorods grown using microwave irradiation in a reaction bath containing 10.0 mM zinc nitrate and hexamethylenetetramine for 10 h; structural defects can be observed especially on the polar face.

**Figure 6:**
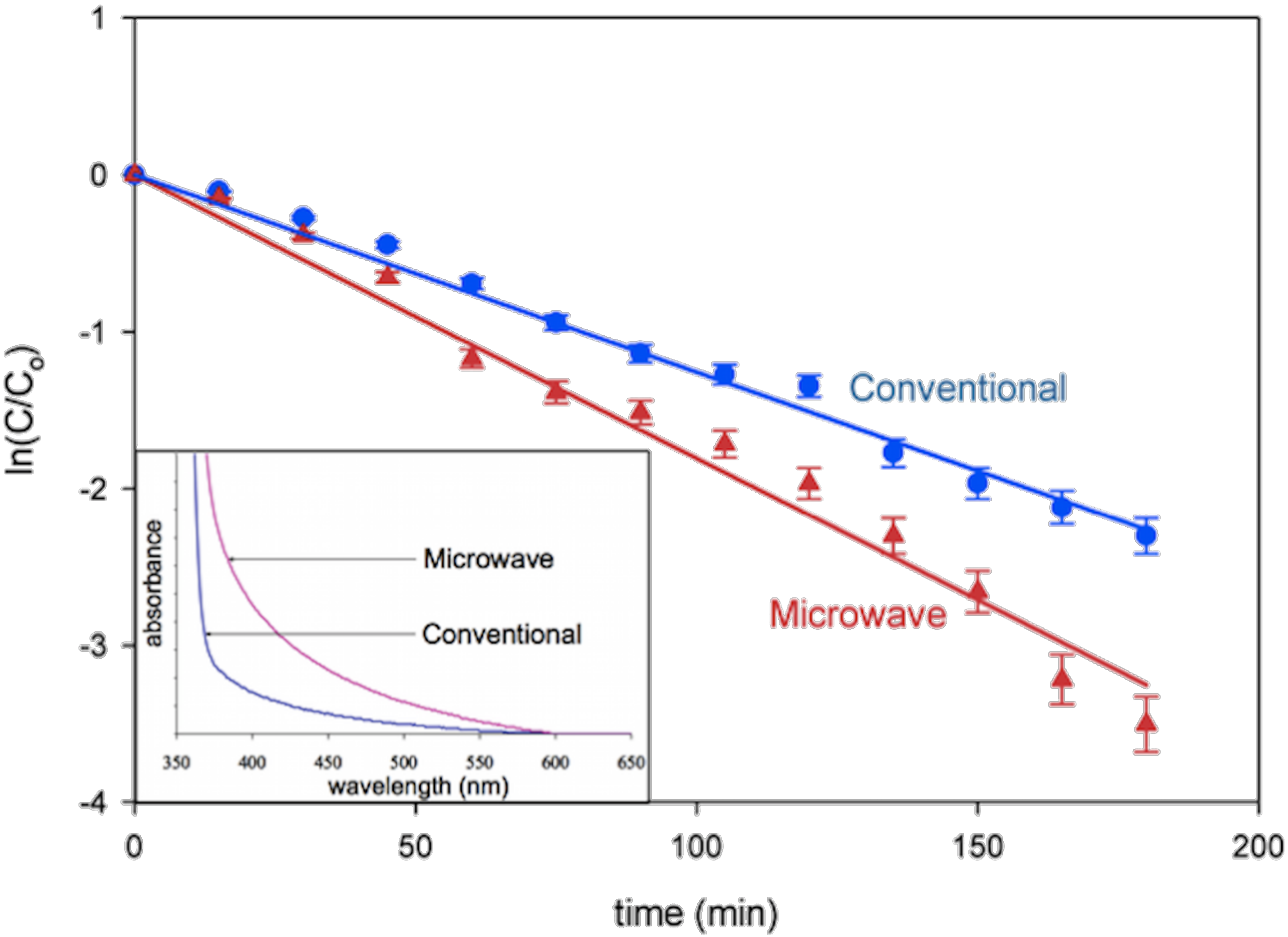
Degradation of methylene blue as a function of ln(*C*/*C*_0_) versus the time of exposure to light in the presence of ZnO nanorods synthesized by the conventional method (Sample 2 with surface area ≈ 47.54 cm^2^) and the fast crystallization method (surface area ≈ 33.74 cm^2^). Inset: UV–vis absorption spectra of ZnO nanorods grown by the conventional hydrothermal method and by microwave irradiation of comparable exposed surface area.

A typical scanning electron micrograph of ZnO nanorods grown through fast crystallization in a reaction bath containing 10.0 mM zinc nitrate hexahydrate and hexamethylenetetramine with microwave irradiation for 10 h is shown in Figure 5. Visible structural defects can be clearly observed on the surface of the rods. In order to further confirm if the fast synthesis route creates increased electron deficient sites, two samples with comparable exposed surface areas, one prepared by the conventional process (Sample 2 with surface area ≈ 47.54 cm^2^) and the other by fast hydrolysis (surface area ≈ 33.74 cm^2^), were selected. Sample 2 was selected as it showed maximum activity compared to Sample 1 and Sample 3. The fast hydrolyzed sample was grown using a reaction bath at a concentration of 10.0 mM of zinc nitrate and hexamine for 4 h under microwave illumination. Optical absorption spectra of the two samples (Figure 6: inset) show higher absorption in the rapidly crystallized nanorods especially in the near UV and visible regions of the electromagnetic spectrum up to about 500 nm. The optical absorptions of both the samples are comparable above 500 nm.

The microwave hydrolyzed nanorods demonstrated better photocatalytic activity (degradation efficiency of 97% in 180 min; *k* = 0.019481 min^−1^) as compared to the conventionally synthesized rods (degradation efficiency of 90% in 180 min; k = 0.012792 min^−1^) (Figure 6). This is attributed to the higher density of electron deficient sites generated during microwave synthesis that can trap photogenerated electrons and reduce recombinations, thereby improving the photocatalytic activity. ZnO nanorods grown through fast crystallization under microwave irradiation not only creates defective crystallites ideal for photocatalysis (Figure 3), but this growth process is also faster compared to the conventional process, thereby saving time and energy during the growth process.

## Conclusion

One-dimensional nanostructures with very high surface to volume ratio can be attractive candidates for photocatalysis. Comparative results of photocatalytic degradation studies on methylene blue with visible light irradiation demonstrated that ZnO nanorods are 12–24% more active than nanoparticulate films. An enhancement of 8% in the photocatalytic activity of ZnO nanorods was achieved through engineered creation of oxygen deficient structures using a fast crystallization process achieved by microwave assisted hydrolysis. This enhancement in the photocatalytic activity was correlated to an increased absorption efficiency of light in the UV and visible regions. Intentional defect inclusion in the crystal of ZnO nanostructures during synthesis is an attractive option for visible light photocatalysis and further results with different pollutants will be presented in succeeding reports. ZnO, apart from having specific structural properties, can also be grown on any type of substrates, such as glass, alumina, polyethylene, polypropylene, steel, cotton, amongst others, through proper seeding and hence is suitable for a large number of applications, e.g., wastewater treatment, etc. that is scalable for practical uses.

## Experimental

### Materials used

Zinc acetate dihydrate [(CH_3_COO)_2_Zn·2H_2_O, Merck], sodium hydroxide [NaOH, Merck], zinc nitrate hexahydrate [Zn(NO_3_)_2_·6H_2_O, APS Ajax Finechem], hexamethylenetetramine [(CH_2_)_6_N_4_, Carlo Erba], ethanol [C_2_H_5_OH, J. T. Baker], and methylene blue [C_16_H_18_N_3_CIS·3H_2_O, Carlo Erba].

#### Synthesis of ZnO nanoparticles

ZnO nanoparticles were synthesized in a colloidal solution with ethanol as the solvent. The co-precipitation technique has been reported in previous publications from our group [19,30]. Briefly, 40 mL of 2 mM zinc acetate solution was heat treated at 70 °C for half an hour. Then 20 mL of 4 mM NaOH solution in deionised water was added and the admixture was hydrolyzed for 2 h at 60 °C.

#### Growth of ZnO Nanorods

The ZnO nanorods were grown hydrothermally on glass substrates, which were initially thiolated for better attachment of the ZnO nanoparticle seeds [31]. Hydrothermal growth of ZnO nanostructures is a simple and thermally efficient process [27]. Seeding was done by dip coating with a colloidal solution of ZnO nanoparticles and annealed at 100 ^o^C for 30 min. The seeds served as nucleation sites and the ZnO nanorods grew preferentially along the c-axis of the wurtzite structure when the seeded substrate was placed in an aqueous chemical bath containing equimolar zinc nitrate hexahydrate and hexamethylenetetramine maintained at 90 °C [31]. Three different growth concentrations were used: (1) Sample 1: 0.1 mM, (2) Sample 2: 1.0 mM and (3) Sample 3: 10.0 mM. The growth of the nanorods was continued for 20 h and the chemical bath was replenished with fresh reactants every five hours to maintain the growth rate [20]. The substrate was then removed and washed several times with deionised water and then annealed at 250 °C for 1 h to remove any unreacted organic deposits. Microwave synthesis of ZnO nanorods on seeded substrates was carried out in a commercial microwave oven operated at the low energy mode for 5 h. Quantification of nanorod size and density were carried out with Scion image processing software on Scanning Electron Microscopy (SEM) obtained from a JEOL JSM-6301F operated at 20 kV.

#### Photocatalysis Tests

Photocatalysis was conducted with a popular test contaminant, methylene blue, which is a heterocyclic aromatic compound, in aqueous solution. Photocatalytic degradation of methylene blue [C_16_H_18_N_3_SCl] (MB) results in the formation of leuco-methylene blue (LMB). A 10 μM solution of MB was prepared in deionised water and put in polymethyl methacrylate (PMMA) cuvettes and a glass slide (3 × 1 cm) with a coating of nanorods (Sample 1, Sample 2 and Sample 3) was placed inside the cuvette with the nanorod surface facing a tungsten-halogen light source (500 W). A glass vessel containing water was placed between the light source and the cuvettes to absorb the UV and infrared light radiated by the lamp. At the sample position, 72 klx of light was measured by a luxmeter calibrated to 550 nm. As a control, a similar glass slide (3 × 1 cm) covered with a thin film of ZnO nanoparticles (diameter ≈ 5–7 nm) by a dip coating process was placed in a cuvette with the nanoparticle side facing the light source. Optical absorption spectra were recorded after different light exposure durations with an Ocean Optics spectrophotometer in order to monitor the rate of decolourisation of the test contaminant. The degradation of the dye was estimated from the reduction in absorption intensity of MB at a fixed wavelength λ_max_ = 665 nm. The degradation efficiency (DE) was then calculated as given in Equation 4:

[4]
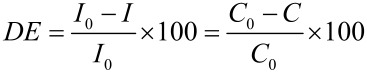


where *I*_0_ is the initial absorption intensity of MB at λ_max_= 665 nm and *I* is the absorption intensity after photoirradiation. *C*_0_ is the initial concentration of the dye and *C* is the concentration after photoirradiation.
